# Supplementary material for: Communication-efficient federated learning via knowledge distillation
Source: Nat Commun. 2022 Apr 19;13:2032. doi: 10.1038/s41467-022-29763-x (PMC9018897; doi:10.1038/s41467-022-29763-x)
Supplement: Supplementary file 1 — Supplementary Information [file 41467_2022_29763_MOESM1_ESM.pdf]

Supplementary Information for "Communication-Efficient Federated Learning via Knowledge Distillation"

Chuhan Wu[1]**, Fangzhao Wu**[2*]**, Lingjuan Lyu**[3]**, Yongfeng Huang**[1*]**, and Xing Xie**[2]

[1]Department of Electronic Engineering, Tsinghua University, Beijing 100084, China
[2]Microsoft Research Asia, Beijing 100080, China
[3]Sony AI, 1-7-1 Konan Minato-ku, Tokyo, 108-0075 Japan
[*]Correspondence: fangzwu@microsoft.com, yfhuang@tsinghua.edu.cn

Supplementary Materials

Datasets

The details of datasets used in our paper are introduced as follows. In the personalized news recommendation task, we use the MIND[1] dataset (available at https://msnews.github.io/) It contains the news impression logs of 1 million users on the Microsoft News platform during 6 weeks. The logs in the last week are used for test, and the rest are for training and validation. The dataset used for adverse drug reaction (ADR) mentioning text detection (denoted as ADR) is released by the 3rd shared task of the SMM4H 2018 workshop[2] (available at https://healthlanguageprocessing.org/smm4h18). The original ADR dataset contains 25,678 samples. However, since many texts in this dataset are no longer available online, we only crawled 16,694 samples for experiments. Following a recent work[3], we use 80% of the dataset for training, 10% for validation and 10% for test. We summarize the detailed statistics of these two datasets (Supplementary Table 1). The datasets for medical named entity recognition (NER) include CADEC[4], ADE[5] and SMM4H[6]. For each of them, we randomly sampled 80% of sentences for training, 10% for validation, and 10% for test. We also provide the detailed statistics of these three datasets (Supplementary Table 1).

Experimental Settings

Hyperparameter Settings

In our experiments, on each client we use the UniLM-Base[7] model as the local mentor. We take pre-trained language model distillation as a representative example in our experiments. We use its submodels with the first 4 or 2 Transformer layers as the mentee models. On the MIND dataset, we incorporate the language model as the news encoder of NAML. On the ADR dataset we apply an attentive pooling and a dense layer after the language model for text classification. The energy thresholds T_{start} and T_{end} are 0.95 and 0.98, respectively. The optimizer we use is Adam[8]. On the MIND dataset, we randomly sample 4 non-clicked news (the number is called negative sampling ratio) for each positive sample to conduct contrastive model training[9]. On the ADR dataset, since the class is highly imbalanced, we use oversampling strategy by repeating positive samples by a certain time (called oversampling ratio). We list the complete hyperparameter settings (Supplementary Table 3). We repeat each experiment 5 times to mitigate occasionality.

Metrics

The metrics used in our experiments are AUC, MRR, nDCG@5 and nDCG@10 on MIND; precision, recall, Fscore on ADR; and Fscore on CADEC, ADE and SMM4H. Note that on the medical NER datasets, the Fscore we use is computed at the entity level, i.e., only extracted entities with both corrected boundaries and type are regarded as correct ones. These metrics are respectively formulated as follows:

$$\text{Precision} = \frac{\text{TP}}{\text{TP}+\text{FP}}, \tag{1}$$

$$\text{Recall} = \frac{\text{TP}}{\text{TP}+\text{FN}}, \tag{2}$$

$$\text{Fscore} = \frac{2*\text{Precision}*\text{Recall}}{\text{Precision}+\text{Recall}}, \tag{3}$$

$$\text{AUC} = \frac{\sum_{\text{p}\in\mathscr{P}}\sum_{\text{n}\in\mathscr{N}} \text{I}[\text{P}(\text{p}) > \text{P}(\text{n})]}{|\mathscr{P}||\mathscr{N}|}, \tag{4}$$

$$\text{MRR} = \frac{1}{\text{N}_\text{p}}\sum_{\text{i}=1}^{\text{N}_\text{p}}\frac{1}{\text{Rank}(\text{p}_\text{i})}, \tag{5}$$

$$\text{nDCG@K} = \frac{\sum_{\text{i}=1}^{\text{K}}(2^{\text{r}_\text{i}}-1)/\log_2(1+\text{i})}{\sum_{\text{i}=1}^{\text{N}_\text{p}} 1/\log_2(1+\text{i})}, \tag{6}$$

where TP, FP and FN are true positive, false positive and false negative, respectively. $P(\cdot)$ is the predicted probability score of a sample to be a positive one. $\mathscr{P}$ and $\mathscr{N}$ respectively denote the sets of positive and negative samples. $I[\cdot]$ is the indicator function. r_i is a relevance score of news with the i-th rank, which is 1 for clicked news and 0 for non-clicked news. Note that nDCG@K is applied to the top K recommendation lists.

Experimental Environment

Our experimental environment is built on a Linux server with Ubuntu 16.04 operation system. The version of Python is 3.6 The server has 4 Tesla V100 GPUs with 32GB memory. The CPU type is Intel(R) Xeon(R) Platinum 8168 CPU @ 2.70GHz. The total memory is 128GB. We use the horovod framework for parallel model training on the 4 GPUs, each of which represents a client.

Model Initialization

In our approach, we use the token embedding layer and the first 4 or 2 layers of UniLM to initialize the mentee model. We do not change the hidden dimension of the model because the UniLMv2 models with other hidden dimensions are not released. Note that our approach does not have limitations on the hidden dimension of the mentee model.

Running Time

On the MIND dataset, the total training of time FedKD_4 and FedKD_2 are take around 66 and 57 hours, respectively. On the ADR dataset, their total training times are about 12 minutes and 10.5 minutes, respectively. On the medical NER datasets, the total training times are 28 and 26 minutes, respectively.

Comparison with Additional Baselines

To provide benchmarks on the datasets used in this work, we compare the performance of our FedKD approach with several baseline methods on these datasets. On the MIND dataset, the additional baseline methods to be compared include: (1) EBNR[10], embedding-based news recommendation with gated recurrent unit (GRU) network; (2) DKN[11], deep knowledge network for news recommendation; (3) NPA[9], news recommendation with personalized attention; (4) NAML[12], news recommendation with attentive multi-view learning; (5) LSTUR[13], news recommendation with long short-term user interest; (6) NRMS[14], news recommendation with multi-head self-attention; (7) FIM[15], fine-grained interest matching for news recommendation. On the ADR dataset, we compare with the following baseline methods: (1) CNN[16], convolutional neural network (CNN) for text classification; LSTM[17], long short-term memory network; (3) CNN+Att[18], using attentive pooling after CNN model; (4) LSTM+Att[19], applying attention pooling to LSTM; (5) MSA[3], a multi-head self-attention based approach for ADR detection; (6) BERT[20], using BERT for ADR detection. On the medical NER datasets, we compare the following methods: (1) CNN-CRF[21], using CNN to capture local contexts and CRF to decode labels; (2) LSTM-CRF[22], using a Bidirectional LSTM network to learn word representations; (3) GRAM-CNN[23], using CNN to capture both character-level and word-level contexts. (4) CNN-LSTM-CRF[24], using CNN to learn character representations and LSTM to learn word representations; (5) S-LSTM-CRF[25], using a stacked Bi-LSTM network for learning word representation; (6) CNN-CLSTM-CRF[26], using a combination of CNN and LSTM to learn word representations in CNN-LSTM-CRF; (7) FedNER[27], a federated NER method for learning NER models from decentralized datasets. The baselines except FedNER are trained on a centralized data storage. From the results on different datasets (Supplementary Table 4, 5 and 6, we find the performance of our FedKD approach consistently outperform all the compared baseline methods (e.g., 70.7% v.s. 68.5% AUC scores on MIND). This is because our approach takes the advantage of the state-of-the-art pre-trained language models and allows the mentor and mentee models to collaboratively learn from each other, which are helpful for learning strong models.

Influence of Client Number

We study the influence of client number on the model performance in this section. We divide the full training data into different numbers of folds to simulate the scenarios with different amounts of labeled data on each client. We show the performance of FedKD and $\text{UniLM}_{4/2}$ under different numbers of clients (Supplementary Figure 1). We find the performance of FedKD is similar and can even be slightly improved when more clients are involved. This is because different mentor models are learned on different data folds, and thereby can encode different views of knowledge. By learning from multiple mentor models on different clients, the mentee model may inherit richer knowledge when more mentor models participate. On the contrary, the performance of $\text{UniLM}_{4/2}$ (Fed) slightly declines with the increase of client number. This may be because the vanilla FedAvg method has some performance sacrifice by learning models for multiple epochs on limited local data.

Impact of Energy Threshold

We then study the influence of the energy threshold T_{start} and T_{end} on the performance and communication cost of our approach. We first vary the value of T_{start} under $T_{\text{end}} = 1$. From the results (Supplementary Fig. 2 (a) and (c)). We find the communication cost is smaller when T_{start} is smaller, while we observe that the performance starts to drop quickly when $T_{\text{start}} < 0.95$. Thus, we chose $T_{\text{start}} = 0.95$ to balance communication cost and model performance. Under $T_{\text{start}} = 0.95$, we then vary T_{end} to compare the performance and communication cost (Supplementary Fig. 2 (b) and (d)). In a similar way, we choose $T_{\text{end}} = 0.98$ to achieve a good tradeoff between model accuracy and communication cost.

Supplementary Figures

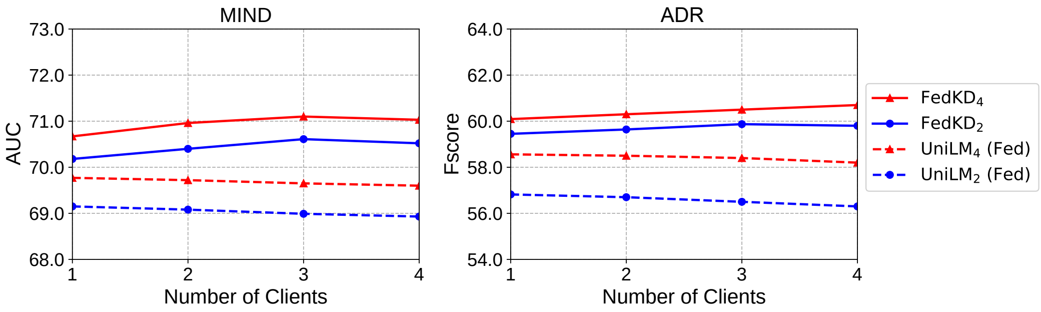

Supplementary Figure 1. Influence of client number on the model performance of FedKD and the vannila federated learning version of UniLM (MIND and ADR datasets). We compare their 4-layer and 2-layer versions. We find that when there are more clients, the performance of vanilla federated models slightly drops, while the performance of FedKD first improves and then decreases. It shows that FedKD is suitable in handling limited local data (but a certain amount of data is still required).

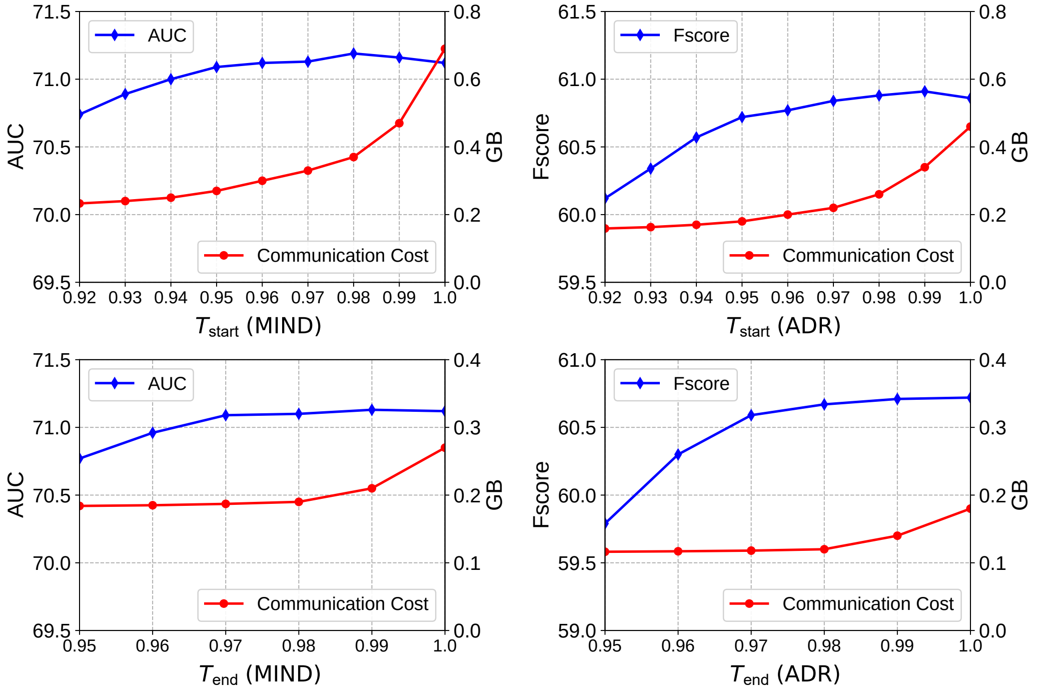

Supplementary Figure 2. Influence of T_{start} and T_{end} on model performance and communication cost (MIND and ADR datasets). The results show that lower energy thresholds usually mean lower communication cost, but the performance scarifice may also be heavier. Thus, we choose $T_{\text{start}} = 0.95$ and $T_{\text{end}} = 0.98$ to balance communication cost and model accuracy.

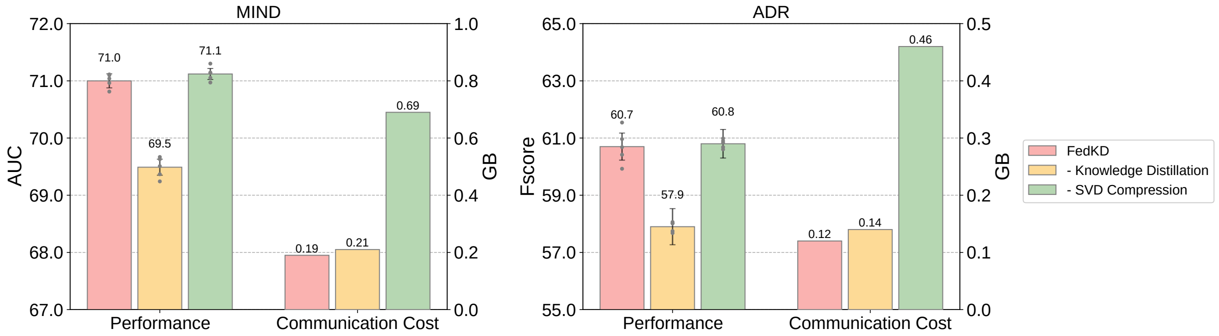

Supplementary Figure 3. Ablation study on knowledge distillation and gradient compression. The mean model performance with 95% confidence intervals (n=5 independent experiments) and communication cost (the cost values are constants) when the knowledge distillation mechanism or the SVD-based compression module is removed. The 4-layer model is used. When there is no knowledge distillation, the student model is used for inference. The results show that knowledge distillation has a major contribution to the model performance, while the SVD compression can greatly decrease the communication cost with minor performance loss.

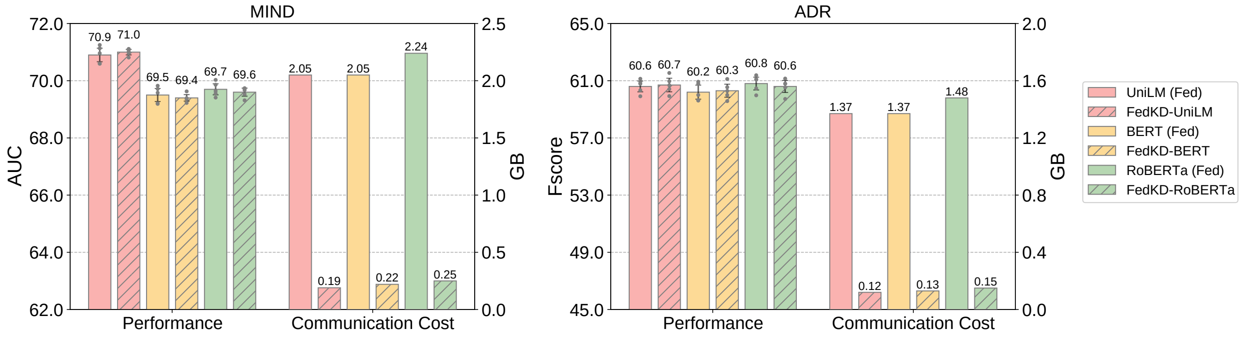

Supplementary Figure 4. Generality of FedKD to different models. Comparison of mean model performance with 95% confidence intervals (n=5 independent experiments) and communication cost (the cost values are constants) when using different basic models, including BERT[28], RoBERT[29], and UniLM[7]. The 4-layer model is used. The results show that FedKD can consistently reduce the communication cost meanwhile retaining high performance.

Supplementary Tables

MIND			
# users	1,000,000	# impressions	15,777,377
# news	161,013	# clicks	24,155,470
avg. title len.	11.52	# training samples	2,186,683
# validation samples	365,200	# test samples	2,341,619
ADR			
# texts	16,694	# positives	1,355
avg. text len.	16.48	# negatives	15,336

Supplementary Table 1. Statistics of the MIND and ADR datasets. MIND is a large scale news recommendation dataset with anonymous user news click logs. ADR is a text classification dataset to identify adverse drug reaction mentioning text. We can see that the classes in ADR are highly imbalanced.

Dataset	# Sentences	Entity Types	# Entities
ADE	4,483	ADE(5678), Drug(5076), Dosage(222)	10,976
C_{ADEC}	7,683	ADE(5937), Drug(1796), Disease(282), Finding(425), Symptom(268)	8,535
SMM4H	3,824	ADE(1707)	1,707

Supplementary Table 2. Statistics of the medical NER datasets. The three datasets have different sizes, number of entities, and entity annotation types. The ADE and C_{ADEC} datasets have multiple entity types and the model need to classify their types.

**Hyperparameters**	MIND	ADR	CADEC/ADE/SMM4H
LM hidden dimension	768	768	768
negative sampling ratio	4	-	-
over sampling ratio	-	2 for LMs, 9 for others	-
attention query dimension	200	200	200
dropout	0.2	0.2	0.2
optimizer	Adam	Adam	Adam
mentor model learning rate	2e-6	1e-5	1e-5
mentee model learning rate	1e-5	3e-5	3e-5
batch size	32	64	64
T_{start}	0.95	0.95	0.95
T_{end}	0.98	0.98	0.98
epoch	3	2	5

Supplementary Table 3. Detailed hyperparameter settings on different datasets. The negative sampling ratio is only used for news recommendation on MIND, and the oversampling strategy is only used for binary classification on ADR.

**Methods**	**AUC**	**MRR**	**nDCG@5**	**nDCG@10**
EBNR	66.1	31.9	34.9	40.5
DKN	65.2	31.5	34.1	39.8
NPA	67.4	32.6	35.5	41.3
NAML	67.4	32.5	35.4	41.2
LSTUR	67.9	33.0	35.9	41.8
NRMS	68.2	33.4	36.3	42.1
FIM	68.5	33.6	36.6	42.4
FedKD_4	**71.0**	**35.6**	**38.9**	**44.8**
FedKD_2	70.5	35.3	38.6	44.3

Supplementary Table 4. Performance of different methods on MIND. The best results (FedKD_4) are in bold. The results show that FedKD can beat many state-of-the-art news recommendation methods.

Methods	Precision	Recall	Fscore
CNN	48.3	52.1	50.2
LSTM	49.6	50.5	50.0
CNN+Att	48.3	53.0	50.5
LSTM+Att	48.5	52.7	50.5
MSA	51.2	53.8	52.4
BERT	56.6	59.8	58.0
$FedKD_4$	**59.4**	**62.8**	**60.7**
$FedKD_2$	58.2	62.4	59.8

Supplementary Table 5. Performance of different methods on ADR. The best results ($FedKD_4$) are in bold. The results show that FedKD outperforms many state-of-the-art ADR mentioning text detection methods.

Model	CADEC	ADE	SMM4H
CNN-CRF	56.5	61.5	31.2
LSTM-CRF	56.8	62.3	31.0
GRAM-CNN	55.9	72.4	26.1
CNN-LSTM-CRF	59.3	73.0	27.7
S-LSTM-CRF	60.6	76.4	32.1
CNN-CLSTM-CRF	60.1	78.8	31.0
FedNER	65.2	82.6	32.7
$FedKD_4$	**67.7**	**87.4**	**34.5**
$FedKD_2$	67.0	85.2	33.9

Supplementary Table 6. Performance of different methods on CADEC, ADE and SMM4H. The best results ($FedKD_4$) are in bold. The results validate the superiority of FedKD in exploiting decentralized datasets for medical NER.

References

1. Wu, F. *et al.* Mind: A large-scale dataset for news recommendation. In *ACL*, 3597–3606 (2020).
2. Weissenbacher, D., Sarker, A., Paul, M. & Gonzalez, G. Overview of the third social media mining for health (smm4h) shared tasks at emnlp 2018. In *SMM4H*, 13–16 (2018).
3. Wu, C. *et al.* Msa: Jointly detecting drug name and adverse drug reaction mentioning tweets with multi-head self-attention. In *WSDM*, 33–41 (ACM, 2019).
4. Karimi, S., Metke-Jimenez, A., Kemp, M. & Wang, C. Cadec: A corpus of adverse drug event annotations. *J. biomedical informatics* **55**, 73–81 (2015).
5. Gurulingappa, H. *et al.* Development of a benchmark corpus to support the automatic extraction of drug-related adverse effects from medical case reports. *J. biomedical informatics* **45**, 885–892 (2012).
6. Weissenbacher, D. *et al.* Overview of the fourth social media mining for health (smm4h) shared tasks at acl 2019. In *SMM4H Workshop*, 21–30 (2019).
7. Bao, H. *et al.* Unilmv2: Pseudo-masked language models for unified language model pre-training. In *ICML*, 642–652 (PMLR, 2020).
8. Bengio, Y. & LeCun, Y. Adam: A method for stochastic optimization. In *ICLR* (2015).
9. Wu, C. *et al.* Npa: Neural news recommendation with personalized attention. In *KDD*, 2576–2584 (2019).
10. Okura, S., Tagami, Y., Ono, S. & Tajima, A. Embedding-based news recommendation for millions of users. In *KDD*, 1933–1942 (ACM, 2017).
11. Wang, H., Zhang, F., Xie, X. & Guo, M. Dkn: Deep knowledge-aware network for news recommendation. In *WWW*, 1835–1844 (2018).
12. Wu, C. *et al.* Neural news recommendation with attentive multi-view learning. In *IJCAI*, 3863–3869 (2019).
13. An, M. *et al.* Neural news recommendation with long-and short-term user representations. In *ACL*, 336–345 (2019).
14. Wu, C. *et al.* Neural news recommendation with multi-head self-attention. In *EMNLP*, 6390–6395 (2019).
15. Wang, H., Wu, F., Liu, Z. & Xie, X. Fine-grained interest matching for neural news recommendation. In *ACL*, 836–845 (2020).
16. Kim, Y. Convolutional neural networks for sentence classification. In *EMNLP*, 1746–1751 (2014).
17. Hochreiter, S. & Schmidhuber, J. Long short-term memory. *Neural computation* **9**, 1735–1780 (1997).
18. Huynh, T., He, Y., Willis, A. & Rueger, S. Adverse drug reaction classification with deep neural networks. In *COLING*, 877–887 (2016).
19. Zhou, X., Wan, X. & Xiao, J. Attention-based lstm network for cross-lingual sentiment classification. In *EMNLP*, 247–256 (2016).
20. Miftahutdinov, Z., Alimova, I. & Tutubalina, E. Kfu nlp team at smm4h 2019 tasks: Want to extract adverse drugs reactions from tweets? bert to the rescue. In *SMM4H*, 52–57 (2019).
21. Collobert, R. *et al.* Natural language processing (almost) from scratch. *JMLR* **12**, 2493–2537 (2011).

22. Habibi, M., Weber, L., Neves, M., Wiegandt, D. L. & Leser, U. Deep learning with word embeddings improves biomedical named entity recognition. *Bioinforma.* **33**, i37–i48 (2017).

23. Zhu, Q., Li, X., Conesa, A. & Pereira, C. Gram-cnn: a deep learning approach with local context for named entity recognition in biomedical text. *Bioinforma.* **34**, 1547–1554 (2017).

24. Ma, X. & Hovy, E. End-to-end sequence labeling via bi-directional lstm-cnns-crf. In *ACL*, 1064–1074 (2016).

25. Lample, G., Ballesteros, M., Subramanian, S., Kawakami, K. & Dyer, C. Neural architectures for named entity recognition. In *NAACL-HLT*, 260–270 (2016).

26. Shen, Y., Yun, H., Lipton, Z., Kronrod, Y. & Anandkumar, A. Deep active learning for named entity recognition. In *RepL4NLP Workshop*, 252–256 (2017).

27. Ge, S. *et al.* Fedner: Medical named entity recognition with federated learning. *arXiv preprint arXiv:2003.09288* (2020).

28. Devlin, J., Chang, M.-W., Lee, K. & Toutanova, K. Bert: Pre-training of deep bidirectional transformers for language understanding. In *NAACL-HLT*, 4171–4186 (2019).

29. Liu, Y. *et al.* Roberta: A robustly optimized bert pretraining approach. *arXiv preprint arXiv:1907.11692* (2019).
