# Supplementary material for: Communication-efficient federated learning via knowledge distillation
Source: Nat Commun. 2022 Apr 19;13:2032. doi: 10.1038/s41467-022-29763-x (PMC9018897; doi:10.1038/s41467-022-29763-x)
Supplement: Supplementary file 2 — Reporting Summary [file 41467_2022_29763_MOESM2_ESM.pdf]

nature portfolio

Corresponding author(s): Yongfeng Huang

Last updated by author(s): Mar 25, 2022

Reporting Summary

Nature Portfolio wishes to improve the reproducibility of the work that we publish. This form provides structure for consistency and transparency in reporting. For further information on Nature Portfolio policies, see our Editorial Policies and the Editorial Policy Checklist.

Statistics

For all statistical analyses, confirm that the following items are present in the figure legend, table legend, main text, or Methods section.

n/a	Confirmed	
☐	☒	The exact sample size (*n*) for each experimental group/condition, given as a discrete number and unit of measurement
☐	☒	A statement on whether measurements were taken from distinct samples or whether the same sample was measured repeatedly
☐	☒	The statistical test(s) used AND whether they are one- or two-sided<br>*Only common tests should be described solely by name; describe more complex techniques in the Methods section.*
☒	☐	A description of all covariates tested
☐	☒	A description of any assumptions or corrections, such as tests of normality and adjustment for multiple comparisons
☐	☒	A full description of the statistical parameters including central tendency (e.g. means) or other basic estimates (e.g. regression coefficient) AND variation (e.g. standard deviation) or associated estimates of uncertainty (e.g. confidence intervals)
☐	☒	For null hypothesis testing, the test statistic (e.g. *F*, *t*, *r*) with confidence intervals, effect sizes, degrees of freedom and *P* value noted<br>*Give P values as exact values whenever suitable.*
☒	☐	For Bayesian analysis, information on the choice of priors and Markov chain Monte Carlo settings
☐	☒	For hierarchical and complex designs, identification of the appropriate level for tests and full reporting of outcomes
☒	☐	Estimates of effect sizes (e.g. Cohen's *d*, Pearson's *r*), indicating how they were calculated

Our web collection on statistics for biologists contains articles on many of the points above.

Software and code

Policy information about availability of computer code

Data collection	No software was used for data collection.
Data analysis	The FedKD and FedAvg algorithms are implemented by the Pytorch 1.5 library. Analysis was performed with the numpy 1.16.5 and Scipy 1.5.2 libraries. Custum code for data analysis are available at https://github.com/wuch15/FedKD. The Python version is 3.6.9. More details can be found in the manuscript methods section and supplementary materials.

For manuscripts utilizing custom algorithms or software that are central to the research but not yet described in published literature, software must be made available to editors and reviewers. We strongly encourage code deposition in a community repository (e.g. GitHub). See the Nature Portfolio guidelines for submitting code & software for further information.

Data

Policy information about availability of data

All manuscripts must include a data availability statement. This statement should provide the following information, where applicable:

- Accession codes, unique identifiers, or web links for publicly available datasets
- A description of any restrictions on data availability
- For clinical datasets or third party data, please ensure that the statement adheres to our policy

The datasets that supports the findings of this study are all available ones, and the use of them in this work adheres to the licenses of these datasets. The MIND dataset is available at https://msnews.github.io/. The ADR dataset is available at https://healthlanguageprocessing.org/smm4h18.The CADEC dataset is available at https://data.csiro.au.The ADE dataset is available at https://sites.google.com/site/adecorpus/. The SMM4H dataset is available at https:// healthlanguageprocessing.org/smm4h19. Source data are provided with this paper.

March 2021

Field-specific reporting

Please select the one below that is the best fit for your research. If you are not sure, read the appropriate sections before making your selection.

☒ Life sciences ☐ Behavioural & social sciences ☐ Ecological, evolutionary & environmental sciences

For a reference copy of the document with all sections, see nature.com/documents/nr-reporting-summary-flat.pdf

Life sciences study design

All studies must disclose on these points even when the disclosure is negative.

Sample size	There are five datasets used in this work and they have different sizes, from thousands of samples to millions of samples. The number of samples are calculated according to the independent data logs with unique sample IDs. The sizes of samples are predefined and provided in the original dataset releases. No special sample selection is conducted.
Data exclusions	We conduct experiments on the raw collected dataset and do not perform any data exclusion operations.
Replication	The MIND dataset splits the data according to the timestamp of samples. The training, validation and test sets of other datasets were randomly selected and there are no overlaps. The results can be replicated by applying the algorithm to each sample in the test set, and can be further validated by conducting experiments on other data with similar formats.
Randomization	Except the MIND dataset that provides different data splits, other datasets were randomly divided into training, validation and test (10%) sets. Each sample was assigned to exactly one of these splits. The MIND dataset is divided by the time of samples and the time periods of different splits have no overlap. The samples in the last week are used for test, the samples in last day before the test set period are used for validation, and the rest samples are used for training. The orders of samples in each set are randomly shuffled.
Blinding	When assigning samples randomly to different sets, researchers were blinded to the sample selection. Research team members were also blinded to the data collection process and all the identities of users and samples had been fully anonymized.

Reporting for specific materials, systems and methods

We require information from authors about some types of materials, experimental systems and methods used in many studies. Here, indicate whether each material, system or method listed is relevant to your study. If you are not sure if a list item applies to your research, read the appropriate section before selecting a response.

Materials & experimental systems

n/a	Involved in the study
☒	☐ Antibodies
☒	☐ Eukaryotic cell lines
☒	☐ Palaeontology and archaeology
☒	☐ Animals and other organisms
☒	☐ Human research participants
☒	☐ Clinical data
☒	☐ Dual use research of concern

Methods

n/a	Involved in the study
☒	☐ ChIP-seq
☒	☐ Flow cytometry
☒	☐ MRI-based neuroimaging
